# The transverse musculocutaneous gracilis flap for autologous breast reconstruction: focus on donor site morbidity

**DOI:** 10.1007/s12282-021-01264-7

**Published:** 2021-06-06

**Authors:** Laura C. Siegwart, Sebastian Fischer, Yannick F. Diehm, Jörg M. Heil, Christoph Hirche, Ulrich Kneser, Dimitra Kotsougiani-Fischer

**Affiliations:** 1 Department of Hand, Plastic and Reconstructive Surgery, Microsurgery, Burn Center, BG Trauma Center Ludwigshafen, Hand and Plastic Surgery, University of Heidelberg, Ludwigshafen, Germany; 2 Department of Surgery, Division of Plastic Surgery, Brigham and Women’s Hospital, Harvard Medical School, Boston, USA; 3 Department of Gynecology, Breast Cancer Center, University of Heidelberg, Heidelberg, Germany

**Keywords:** TMG, Transverse musculocutaneous gracilis flap, TUG, Transverse upper gracilis flap, Donor site morbidity, Breast reconstruction

## Abstract

**Purpose:**

The transverse musculocutaneous gracilis (TMG) flap is as a valuable alternative in autologous breast reconstruction. The purpose of this study was to evaluate the donor site morbidity and secondary refinement procedures after TMG flap breast reconstruction.

**Methods:**

A retrospective study was conducted, including all patients who received TMG flap breast reconstructions, from January 2012 to August 2019. Primary outcomes were surgical site complications of the donor site and secondary refinement procedures carried out for aesthetic or reconstructive purposes for the medial thigh. Secondary outcomes of interest were lipofilling procedures for optimization of the reconstructed breasts.

**Results:**

Ninety-nine patients received 159 TMG flaps for breast reconstruction. Patients’ mean BMI was 23.5 (15.6–32.5) kg/m^2^. Bilateral breast reconstructions were performed in 60.6%. The mean flap volume was 330 (231–440) g. Surgical site complications occurred in 14.5% of the TMG donor sites and wound dehiscence was the most common complication (9.4%). Lymphedema occurred in 1.8% of the donor thighs. Aesthetic refinement procedures were performed in 25.2% on the donor thigh or contralateral thigh. Secondary lipofilling was performed in 54.1% of the reconstructed breasts and fat was harvested in only 11.9% from the legs.

**Conclusion:**

The TMG flap breast reconstruction combines low donor site morbidity with adequate volume for appealing breast results, particularly in slim-to-normal weight patients. However, patients should be informed about the likelihood of secondary refinement procedures on the donor site and the need of lipofilling to optimize the breast shape and volume.

## Introduction

Currently the lower abdomen represents the primary donor site for autologous breast reconstruction [1]. Based on the individual anatomy the deep inferior epigastric artery perforator (DIEP) flap or its muscle-sparing equivalent the muscle-sparing 2 transverse rectus abdominis (MS2-TRAM) flap are considered to be the gold standard [2–4]. However, due to inadequate tissue availability, previous surgeries or patient’s preference, surgeons might choose alternative donor sites. The medial thigh is a valuable option for soft tissue harvest [5–9]. First introduced in 1992 by Yousif et al., the transverse musculocutaneous gracilis (TMG) flap exploits the excess of fat and skin laxity of the medial thigh to reconstitute breast volume [10, 11]. The reliable anatomy of the nutrient pedicle, the medial circumflex artery, and supine patient position allow for a safe and straight-forward two-team approach. In Europe Schoeller et al. and Wechselberger et al. popularized the use of the TMG flap in patients who do not qualify for the abdominal donor site [6, 12]. In addition to appealing outcome of the reconstructed breast, the TMG flap has been advertised as a welcome opportunity to provide a medial thigh lift on the donor site. However, the literature regarding the donor site morbidity after TMG flap breast reconstruction is scarce and controversial. Few studies have evaluated the outcome of the medial thigh donor site in detail and limitations exist due to small patient samples of the presented studies and incomplete presentation of donor site outcome measures [7, 8, 13–15]. Craggs et al. reported substantial donor site complications in 59% of the patients [13]. Fattah et al. reported wound complications in 36.8% of the donor thighs [16]. In contrast, Pülzl et al. outlined excellent donor site outcomes concerning thigh symmetry and thigh contour in 42% and 26% of the patients, although labial stretching was noted in 11.1% of the patients [14]. The limited skin and soft tissue availability on the medial thigh resulting in small breast volume has been listed as another shortfall of the TMG flap breast reconstruction [13, 17].

In this article we present our experience with TMG flap breast reconstruction, with particular emphasis on donor site morbidity of the medial thigh. In addition, we evaluate secondary refinement procedures of the reconstructed breast and review technical refinements of TMG flap harvest and donor site closure.

### Patients and methods

## Study design and data acquisition

Following local ethics committee approval [2018-13902_1], a retrospective study was conducted, including all patients who had received TMG flaps for autologous breast reconstruction, between January 2012 and August 2019 in our breast reconstruction center. The protocol of this study was in accordance with the Declaration of Helsinki and its later amendments.

The postoperative follow-up was at least six months after TMG flap breast reconstruction with regularly clinical follow-up visits.

The electronic inpatient hospital system and patient charts were used for data acquisition. We extracted patient characteristics such as gender, age, body mass index (BMI in kg/m^2^), smoking status, comorbid conditions, past medical history, including radiation and chemotherapy, and previous abdominal operations. Also, intra-operative data of TMG flap harvest and donor site closure were gathered (operation time, unilateral vs. bilateral reconstruction, timing of reconstruction, TMG flap size and weight and technical refinements in TMG flap harvest) as well as the number and character of secondary procedures on the donor site and the breast.

Primary outcomes of interest were non-operative and operative surgical site complications of the medial thigh in short-term such as wound dehiscence, seroma, hematoma and wound infection and secondary refinement procedures for aesthetic and non-aesthetic purposes of the donor thigh and contralateral thigh. Prognostic risk factors for operative and non-operative surgical site complications were evaluated. Secondary outcomes of interest were lipofilling procedures for optimization of the reconstructed breasts. Moreover, technical refinements of the TMG flap harvest procedure and donor site closure were reviewed.

### Surgical technique of TMG flap harvest and donor site closure

**Fig. 1:**
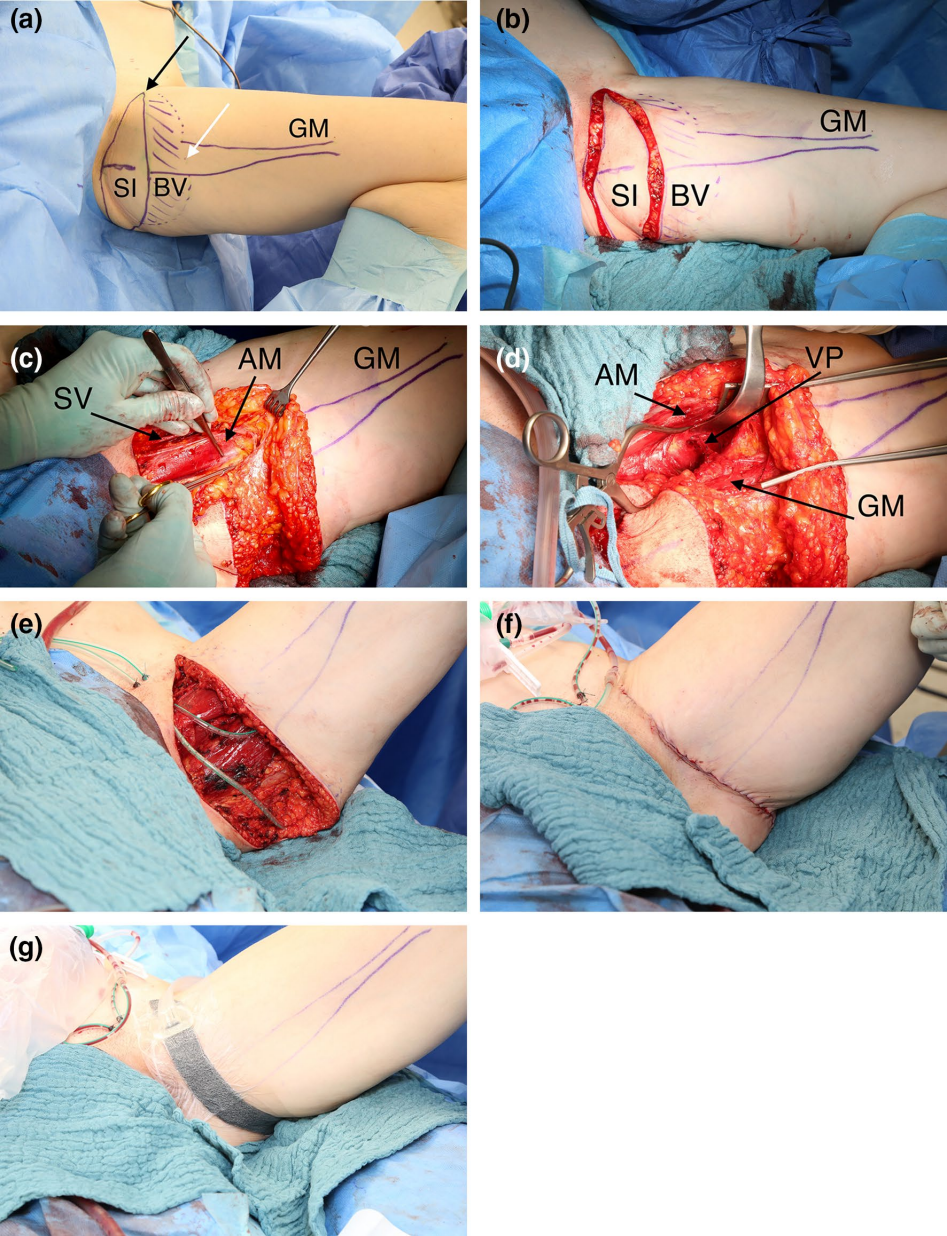
**a** Thirty seven year old patient (BMI 20.7 kg/m^2^) with implant failure of the right reconstructed breast after skin sparing mastectomy due to breast cancer. Patient is in supine position. Left leg in frog position with preoperative markings of the transverse musculocutaneous gracilis (TMG) flap in average flap dimension outlining the proximal skin island (SI), the subcutaneous fat extension to boost flap volume [beveling (BV)] and the gracilis muscle (GM). The skin island is limited to the medial aspect by the neurovascular bundle (black arrow) and to the inferior aspect by pinch grip (white arrow). **b** Circumferential incision of the TMG flap skin island. **c** Opened muscle fascia following complete soft tissue preparation of the TMG flap sparing the saphenous vein (SV) and lymphatic collectors. The vascular pedicle to the gracilis muscle is visualized between the adductor longus muscle (AM) and the gracilis muscle. **d** Preparation of the vascular pedicle (VP) in the septocutaneous space below the retracted adductor longus muscle to its origin from the medial circumflex artery. **e** Open donor site following complete lift of the TMG flap from the medial thigh. **f** Multiple layer closure of the TMG donor site on the medial thigh. **g** Closed incision negative pressure therapy on the TMG donor site

Pre-operative markings were made in a standing position. First, the femoral neurovascular bundle was identified, defining the anterior aspect of the TMG flap and the posterior aspect was marked shortly before the midline of the dorsal thigh. Second, the superior border of the TMG flap was set in the gluteal fold. Third, the inferior border of the skin island was designed using a pinch grip. In the operating room the patient was placed in frog leg position (Fig. 1a). Superficial dissection of the subcutaneous tissue was performed from the anterior aspect of the thigh until the saphenous vein and lymphatic collectors were located (Fig. 1b). Subsequently, deep dissection was performed to include the full thickness of adipose tissue. As needed, subcutaneous fat, located inferiorly from the TMG skin island was included to extend flap volume. Following soft tissue preparation, the muscle fascia was incised and the soft tissue elevated of the semi-membranosus and semi-tendinosus muscle until the adductor magnus muscle and the gracilis muscle were visible (Fig. 1c). The vascular pedicle to the gracilis muscle was thoroughly prepared in the septocutaneous space to its origin from the medial circumflex artery (Fig. 1d). Side branches to the adductor longus muscle were previously clipped. The gracilis muscle was prepared and cut as distally as possible. Care was taken to coagulate or ligate the minor vascular pedicles. The vascular pedicle was transected and subsequently the TMG flap was elevated from the medial thigh (Fig. 1e). Two suction drains were inserted. In some patients suspension of the superficial fascial system to the pubic bone was performed based on the surgeons preference using inverted single button sutures (Vicryl^®^, size 0, Ethicon, Norderstedt, Germany), adopted from cosmetic thighplasty. Donor site closure was achieved in three layers starting with dead space reduction by taking a few big bites of the deep soft tissue. Inverted single button sutures were used for subcutaneous closure (Monocryl 2.0, Ethicon Norderstedt, Germany) followed by a continuous intracutaneous suture with resorbable material (Monocryl 3.0, Ethicon Norderstedt, Germany). Patients received a self-made closed incision negative pressure therapy (CINPT) system (Smith & Nephew GmbH, Hamburg, Germany) or sterile adhesive strips (Leukosan Strip, BSN Medical, Hamburg, Germany) (Fig. 1f). Postoperative compression garments for the abdomen were worn for up to 3 months.

Mobilization was started on the first postoperative day. Drain removal was performed when the maximal output rate sunk below 30 ml/24 h.

### Statistics

Data are presented as frequencies for categorical variables. Means and range were used for continuous variables. We performed univariate logistic regression analyses to evaluate prognostic risk factors (age, BMI, TMG flap width, beveling) associated with non-operative and operative surgical site complications. The performance of the model was based on the Hosmer–Lemeshow test. The Odds ratio β1 (OR) and its 95% confidence interval (CI) were calculated for each independent prognostic risk factor. *P* values ≤ 0.05 were considered statistically significant.

All data analyses were performed by Prism 8.3.0 software (GraphPad Software, California, USA).

## Results

In total, the study evaluated 159 TMG flap breast reconstructions performed in 99 female patients. Patients’ mean age was 42.0 (22–66) years and the BMI was on average 23.5 (15.6–32.5) kg/m^2^. Most of the patients included showed normal weight (61.6%; BMI 18.5–24.9 kg/m^2^). Twenty-eight percent of the patients were overweight (28.3%; BMI 25.0–29.9 kg/m^2^), while only 5% of the patients were obese (5%; BMI ≥ 30.0 kg/m^2^). Five percent of the patients receiving TMG flap breast reconstruction were even underweight (5%; BMI < 18.5 kg/m^2^).

Breast cancer was the most frequent cause for breast reconstruction (82.8%). Forty-eight percent (47.5%) of the patients carried breast cancer predisposition genes BRCA1 or BRCA 2. Sixty percent (60.6%) of the patients received bilateral breast reconstructions. Bilateral breast reconstructions were usually performed in two surgeries (98.7%). The lag between the two surgeries averaged 3.6 (1.4–18.8) months.

Patients’ characteristics are shown in Table 1.

**Table 1:** Patients’ characteristics

Patients	*N* = 99
Age (years), M (range)	42 (22–66)
BMI (kg/m^2^), M (range)	23.5 (15.6–32.5)
BMI (kg/m^2^), (*N*, %)	
< 18.5	5 (5.0%)
18.5–24.9	61 (61.6%)
25–29.9	28 (28.4%)
30–34.9	5 (5.0%)
Diabetes mellitus (*N*, %)	1 (1.0%)
Coagulation disorder (*N*, %)	5 (5.1%)
Active smoker (*N*, %)	22 (22.2%)
Preoperative chemotherapy (*N*, %)	51 (51.5%)
Preoperative radiation (*N*, %)	53 (53.5%)
Indication for breast reconstruction (*N*, %)	
Therapeutic mastectomy due to breast cancer	82 (82.8%)
Idiopathic	49 (49.5%)
BRCA1 or BRCA2 gene mutation	33 (33.3%)
Prophylactic mastectomy (BRCA1 or BRCA2)	14 (14.2%)
Poland syndrome (*N*, %)	1 (1.0%)
Breast aplasia (*N*, %)	1 (1.0%)
Mastopathy (*N*, %)	1 (1.0%)
Reconstruction laterality (*N*, %)	
Unilateral	39 (39.4%)
Bilateral	60 (60.6%)

*N* number, *M* mean, *BMI* body mass index

**Fig. 2:**
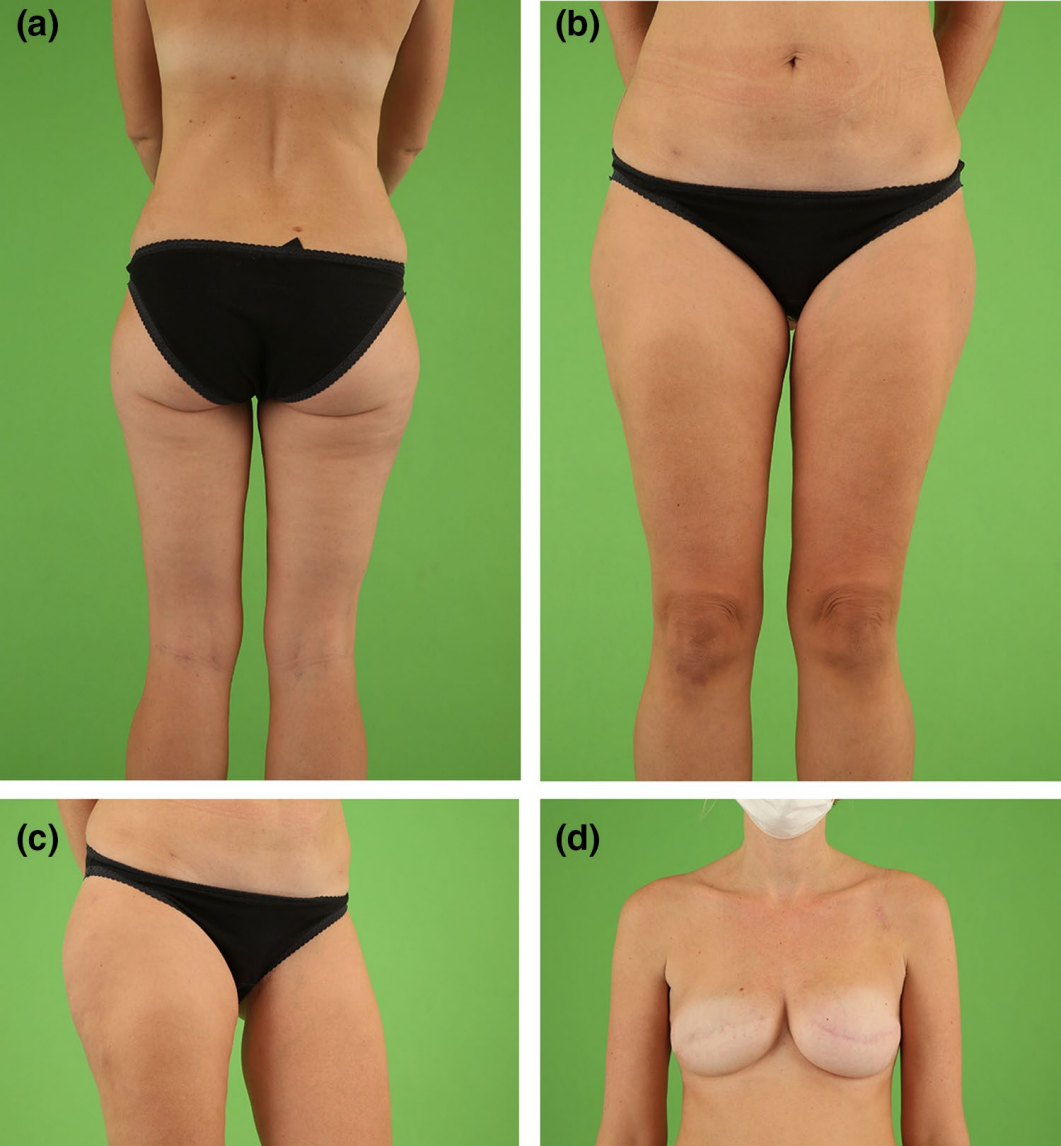
Normal weight female patient (37 years, BMI 20.7 kg/m^2^) with positive BRCA mutation status and invasive ductal carcinoma on the right breast in the medical history. Salvage reconstruction of the right breast with TMG flap from the left thigh following implant failure after skin sparing mastectomy and immediate silicone implant reconstruction on both sides. Skin sparing mastectomy and immediate silicone implant reconstruction of the left breast. The patient had one refinement surgery on the left donor thigh to enhance the contour. Two procedures of lipofilling of the right TMG flap breast were performed, one combined with the refinement surgery of the donor thigh and one combined with the excision of the TMG skin island on the right breast. Postoperative view at 2.0-year follow-up. **a** Back view with concealed donor site scar in the natural crease of the left thigh. **b** Front view with natural symmetry of the thighs after unilateral TMG flap harvest with concealed donor site scar in the groin of the left donor thigh. **c** Flexed left donor thigh with inconspicuous scar in the groin. **d** Excellent shape with natural symmetry of both moderate size breasts following TMG flap reconstruction of the right breast and silicone implant reconstruction of the left breast

**Fig. 3:**
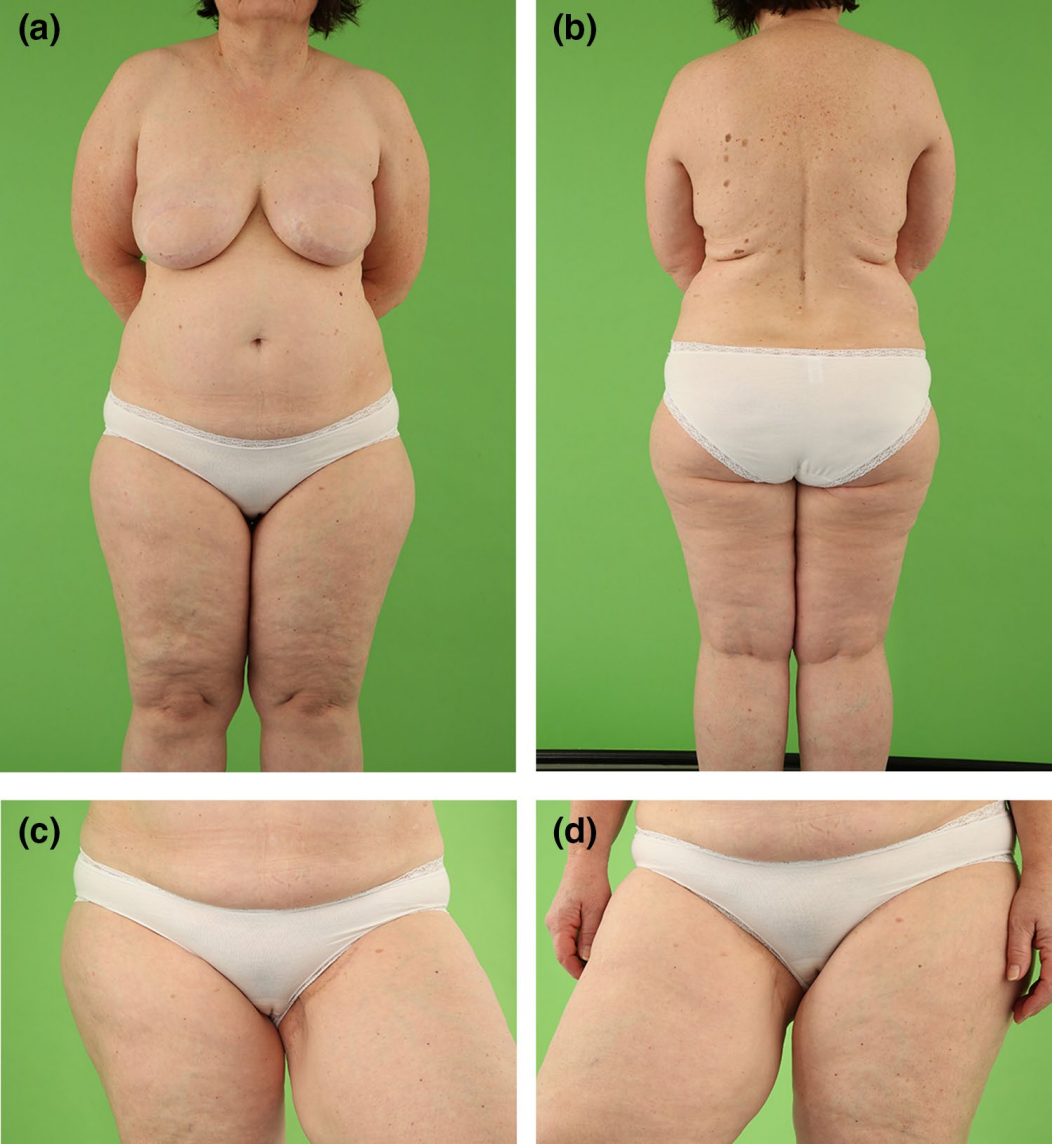
Overweight female patient (51 years, BMI 29.8 kg/m^2^) with positive BRCA mutation status and ductal carcinoma in situ on the right breast and invasive ductal carcinoma on the left breast in the medical history. Bilateral skin-sparing mastectomy and immediate TMG flap breast reconstruction in two separate surgeries after successful breast cancer therapy. The patient had one procedure of lipofilling per breast. Postoperative view at 5.2-year follow-up. **a** Front view with natural symmetry of both reconstructed large size breasts following TMG flap breast reconstruction. Inconspicuous skin color of the TMG skin islands on both reconstructed breasts. Concealed donor site scars in the groin of both donor thighs. **b** Back view with concealed donor-site scars in the natural crease. **c** Left donor thigh with inconspicuous scar in the groin. **d** Right donor thigh with inconspicuous scar in the groin

The most common indication to choose the TMG flap for autologous breast reconstruction was a slim body paired with inadequate tissue availability on the lower abdomen (76.0%) (Fig. 2a–d). Sixty-seven percent (66.6%) of the patients had a BMI < 25.0 kg/m^2^. However, 33.4% of the patients who received TMG flap breast reconstruction had a BMI ≥ 25.0 kg/m^2^ (Fig. 3a–d). Other indications for the TMG flap included previous surgeries (15.0%) or inadequate anatomical preconditions on the abdomen (4.0%) or patients’ preference (5.0%). The various indications for TMG flap breast reconstruction are summarized in Table 2.

**Table 2:** Indications for TMG flap breast reconstruction

Patients	*N* = 99
Slim or regular body shape (*N*, %)	76 (76.0%)
Previous abdominal surgery (*N*, %)	15 (15.0%)
Visceral surgery	8 (8.0%)
Cosmetic abdominoplasty	4 (4.0%)
DIEP flap harvest	3 (3.0%)
Anatomical preconditions (*N*, %)	4 (4.0%)
Abdominal hernia (*N*, %)	2 (2.0%)
Rectus diastasis (*N*, %)	1 (1.0%)
Inadequate abdominal perforator (*N*, %)	1 (1.0%)
Patient’s preference (*N*, %)	5 (5.0%)

*N* number

TMG flap breast reconstructions were performed equally as immediate (52.2%) or delayed (47.8%) procedures. Salvage procedures due to implant failure (17.6%) or prior flap loss (3.1%) were conducted in 20.7% of the breasts. The mean operation time for unilateral TMG flap breast reconstruction averaged 253 (154–553) minutes. The mean flap size was 20.3 (14–27) cm × 7.2 (5.5–10) cm and the mean flap weight was 330 (231–440) g. Surgical refinements in TMG flap harvest and donor site closure included beveling of subcutaneous tissue inferior to the TMG skin island to increase TMG flap volume (89.3%), suspension of the superficial fascial system (scarpa fascia) to the pubic bone (23.9%) as well as the use of closed incision negative pressure therapy on the closed incision line (5.0%). The overall TMG flap success rate was 97.5%. All details of the intra-operative procedures are listed in Table 3.

**Table 3:** Intra-operative characteristics

TMG flap breast reconstructions	*N* = 159
TMG flap breast reconstruction (*N*, %)	
Immediate	83 (52.2%)
Delayed	76 (47.8%)
Salvage procedures (*N*, %)	33 (20.7%)
Implant failure	28 (17.6%)
Flap loss	5 (3.1%)
TMG flap weight (g), M (range)	330 (231–440), (*N* = 10)
TMG flap length (cm), M (range)	20.3 (14–27), (*N* = 44)
TMG flap width (cm), M (range)	7.2 (5.5–10.0), (*N* = 85)
Operation time (minutes), M (range)	253 (145–553)
Surgical refinements donor site (*N*, %)	
Inferior beveling of subcutaneous tissue	142 (89.3%)
Suspension of the superficial fascial system to the pubic bone	38 (23.9%)
Closed incision negative pressure therapy	8 (5.0%)
Flap success	155 (97.5%)

*N* number, *M* mean

The incidence of non-operative surgical site complications such as delayed wound healing (6.9%), minor seroma (3.1%) and minor wound infection (1.9%) on the medial thigh was 11.9%. Non-operative surgical site complications were managed in the outpatient clinic with the exception of wound infections. Operative surgical site complications of the donor site occurred in 14.5% of the donor thighs. The most common reason to take a patient back to the operation room was wound dehiscence (9.4%), followed by seroma (2.5%).

Univariate logistic regression analysis revealed no prognostic risk factors associated with non-operative and operative surgical site complications [BMI: OR = 1.00, CI (0.90–1.11), *p* = 0.95; age: OR = 1.02, CI (0.98–1.06), *p* = 0.26; TMG flap width: OR = 1.26, CI (0.71–2.22), *p* = 0.41; beveling OR = 0.78, CI (0.27–2.60), *p* = 0.66 and suspension to pubic bone OR = 0.59, CI (0.22–1.41), *p* = 0.26].

The mean follow-up time was 42 (6–91) months. Secondary refinement procedures were performed in 25.2% of the donor thighs to optimize the aesthetic outcome of the operated or contralateral thigh. The most common procedure on the TMG donor site was scar correction (10.1%), followed by dog ear resection (8.8%) and contour alignment by liposuction (2.5%). An alignment of the contralateral thigh was performed in 3.8% by liposuction or thigh lift. Ninety-three percent (92.6%) of the refinement surgeries of the donor site were combined with breast touch-up procedures such as fat grafting, nipple-areola reconstruction or contralateral mastopexy. All fat grafts gained by liposuctions for alignment purposes were used for lipofilling of the breast. Non-aesthetic secondary procedures such as skin harvest for nipple areola complex (NAC) reconstructions were performed in 5.0% of the TMG donor sites. Lymphedema evolved in 1.8% of the donor thighs. Labial spreading was not present in our study population.

In addition, on average 1.6 procedures of lipofilling were performed in 54.1% of the reconstructed breasts to enhance the contour, shape or volume. Liposuction for fat harvest from the donor thigh or contralateral thigh was performed in only 11.9% of the procedures. In contrast, the abdomen, flanks and outer thighs were used for fat harvest in 53.5%, 20.2% and 14.4%, respectively. On average 101 ml (range 20 ml–330 ml) of pure fat was injected per lipofilling procedure.

Details of outcome measures on the TMG donor site are listed in Table 4.

**Table 4:** Outcome measures on the TMG donor site

TMG donor sites	*N* = 159
Surgical site complications donor sites, total (*N*, %)	42 (26.4%)
Non-operative surgical site complications (*N*, %)	19 (11.9%)
Delayed wound healing	11 (6.9%)
Seroma	5 (3.1%)
Wound infection	3 (1.9%)
Operative surgical site complications (*N*, %)	23 (14.5%)
Wound dehiscence	15 (9.4%)
Seroma	4 (2.5%)
Wound infection	2 (1.3%)
Hematoma	2 (1.3%)
Aesthetic refinements donor site, total (*N*, %)	40 (25.2%)
Scar correction	16 (10.1%)
Dog ear excision	14 (8.8%)
Contour alignment (liposuction)	4 (2.5%)
Contralateral thigh alignment (lift/liposuction)	6 (3.8%)
Non-aesthetic secondary procedures donor site (*N*, %)	
Skin harvest for NAC reconstruction	8 (5.0%)
Lymphedema donor site	3 (1.8%)

## Discussion

Our results substantiate that the TMG flap is a suitable choice in unilateral and bilateral breast reconstruction in slim and normal weight patients. The TMG flap presents low donor site morbidity on the medial thigh while providing adequate volume to restore small to moderate size breasts. Beveling of subcutaneous tissue inferior to the TMG skin island is a safe option to increase the volume of the TMG flap without increasing surgical site complications. However, lipofilling is needed in about 54.1% of the reconstructed breasts to optimize the aesthetic outcome. Moreover, secondary refinement procedures on the donor site are not uncommon.

Autologous breast reconstruction in thin patients is often challenging due to an apparent lack of adequate donor sites. Previous studies indicated TMG flap breast reconstruction to be more likely performed in slim and normal weight patients compared with those having abdominal-based breast reconstruction [6, 13, 18, 19].

This was confirmed in our study, with the majority (66.6%) of patients receiving TMG flap breast reconstruction having a BMI < 25.0 kg/m^2^ and having insufficient tissue bulk on the lower abdomen. Notably, one-third (33.4%) of the patients had a BMI > 25 kg/m^2^ and qualified also for TMG flap due to other selection criteria. In these patients, the specific body and fat distribution type with excess tissue on the thighs influenced the choice of free flap towards the TMG flap. Significant scars on the abdomen presented another exclusion criterion for abdominal-based free flap breast reconstruction [6, 18]. However, an adequate TMG flap volume with a mean flap weight of 330 g could be harvested from the medial thigh donor site in our patients, where the proportional excess of soft tissue can be significant compared to other parts of the body [20]. In this context Weichman et al. investigated the impact of low BMI on the feasibility of performing autologous breast reconstruction with various flaps from the lower abdomen, bottom and thigh [19]. Similar to our study, the mean flap weight per breast used was 387 g in low–normal (18.5–22 kg/m^2^) weight patients and 367 g in high–normal (22–25 kg/m^2^) weight patients. This flap weight was sufficient to provide body appropriate breast reconstruction in that particular patient population. The aforementioned study supports the experience of successful tissue harvest in our patient population and emphasizes the suitability of TMG flap breast reconstruction in slim and normal weight patients. Moreover, secondary lipofilling is a good option to increase breast volume. However, in patients with large breasts reliable alternatives such as double TMG flaps for unilateral breast reconstruction should be considered [21].

Proper donor site selection is key to achieve excellent results of both the reconstructed breast and donor site. Inadequate soft tissue availability and tight wound closure might result in surgical site complications with inferior scar formation or contour irregularities on the donor site. In our study operative surgical site complications were present in 14.9% of the donor sites. Furthermore 11.9% of the operated thighs showed non-operative surgical site complications, which could be managed in the outpatient clinic. Also, Vollbach et al. outlined 13.2% non-operative complications on the medial thigh donor site [22]. In comparison, surgical site complications on the lower abdomen donor site are reported to be as high as 33% in unilateral DIEP flap breast reconstruction and 31% in bilateral DIEP flap breast reconstruction in slim to normal weight patients (BMI < 25.0 kg/m^2^), similar to the complication rate of high-risk patients with obesity [23, 24]. Notably, the average DIEP flap volume per breast was similar to TMG flap volume in bilateral breast reconstruction [mean 365 (78–654) g] [23].

Turning to the discussion of secondary refinement procedures, a considerable number of patients in our study received refinement procedures on the donor thigh or contralateral thigh (25.2%). Twenty-two percent of the donor thighs were optimized due to aesthetic complaints and 2.5% of the contralateral thighs were operated to optimize the symmetry after unilateral TMG flap harvest. Notably, 92.6% of the refinement procedures were combined with breast touch-up procedures such as lipofilling and nipple-areola reconstruction of the reconstructed breast or mastopexy of the contralateral breast. The most common refinement procedures were scar correction due to scar lowering, scar widening or painful scarring (10.1%) and dog ear excision (8.8%). Liposuction of the donor thigh or contralateral thigh was rarely performed in order to correct the thigh contour or asymmetries (6.3% of the thighs). Surprisingly, liposuction of other parts of the body such as the abdomen (53.5%), flanks (20.2%) and outer thighs (14.4%) was more commonly performed to harvest fat for lipofilling of the reconstructed breasts. The number of secondary refinement procedures in our study is in line with that of the few previous studies. Nickl et al. performed liposuction for harmonization of the contralateral thigh in 16.2% of the thighs and Wechselberger et al. conducted scar correction in 27% of the donor sites [8, 17, 25].

However, the need for refinement procedures on the TMG donor site is lower compared to that on the lower abdomen donor site in autologous breast reconstruction. In a recent systematic review, Lindenblatt et al. summarized the likelihood of unpleasant donor site results in DIEP flap breast reconstruction, not at least due to the use of this popular microsurgical procedure in unsuitable patients [26]. In this context Enajat et al. described a refinement rate of 44.5% after DIEP flap or superficial inferior epigastric artery (SIEA) flap harvest to enhance the aesthetic outcome on the abdominal donor site, which was confirmed by Niddam et al. [27, 28]. Weitgasser et al. described a lower donor site morbidity on the medial thigh in a recent cohort study comparing double DIEP flaps to double TMG flaps in simultaneous bilateral breast reconstructions [18]. They demonstrated that 23.7% of double DIEP patients had donor site complications. In contrast only 16.3% of double TMG patients showed donor site complications (*p* = 0.90) with no functional impairments such as abdominal wall weakness or hernia. However, postoperative lipofilling of the breast was more often performed in double TMG patients (65.1% vs. 38.2%, *p* < 0.05). In our study, on average 1.6 procedures of lipofilling were necessary in 54.1% of the reconstructed breasts to optimize the contour, shape or volume of the breast. For reference, the costs of secondary refinement procedures are usually covered by the health insurance in Germany, which may also explain the moderate to high rate of secondary refinement procedures in our study. However, Russe et al. also reported 1.8 fat grafting procedures in 59% of the patients to enhance the aesthetic outcome rather than only boosting the volume after TMG flap breast reconstruction in a multicenter study conducted in Austria and Germany [9].

There is an increasing desire for postmastectomy breast reconstruction in the Asian population, with autologous reconstruction accounting for 49% of the procedures [29, 30]. To date, the DIEP flap is the most popular choice for autologous breast reconstruction [31]. However, Asian women represent ethnic differences, including a low BMI with few redundant abdominal tissues, small breast size, and a disposition for hypertrophic scarring [32, 33]. In light of these physical preconditions, the TMG flap may present a first-line option for breast reconstruction in the Asian population with reduced donor site morbidity compared to abdominal-based free flap breast reconstruction.

Until now, few recommendations for surgical refinement of the TMG donor site existed [6, 8, 34]. Based on the authors’ experience and a thorough review of the literature, donor site morbidity can be minimized with accurate patient selection, avoidance of oversized flap dimensions and compliance to the anatomy. Soft tissue excess should be evaluated with pinch grip in abduction to allow for easy donor site closure. In case of tension, flap dimensions should be reduced to avoid unfavorable scarring and labial spreading. Instead the inclusion of fat distal of the TMG skin islands, the so-called beveling, allows for safe volume maximization. However, to include the perforators that arise from the gracilis muscle and supply the skin island and subcutaneous tissue, the TMG height should not go below 6 cm [7]. The low rate of lymphedema in our patient population (1.8% of the donor thighs) is related to the careful TMG harvest, respecting the anatomy of the vein and lympathic system of the thigh. To avoid lymphedema the anterior flap border pointed out by the femoral neurovascular bundle should not be exceeded, and during dissection of the saphenous vein lymph collectors proximate below should be preserved [7, 34]. Also flap extension should not go beyond the posterior thigh midline to prevent posterior flap tip necrosis, but should be as close as possible to the midline [15]. In addition, suspension of the inner thigh tissue against the pubic periosteum, adopted from cosmetic thighplasty may prevent the sagging of the thigh and labial spreading, [8, 13]. Moreover, closed incision negative pressure therapy may reduce the number of surgical site complications on the donor site, as we have shown previously in abdominal based breast reconstruction [35].

The limitations of the present study relate to its retrospective design. There could be unrecorded complications due to incomplete documentation. However, the review of the medical records was conducted with high sensitivity and precision. In addition, some questions remain unanswered regarding functional aspects such as loss of sensitivity and muscle strength of the donor thigh. Also, patient-reported outcome measures should be surveyed to explore the impact of the medial thigh donor site on daily living and quality of life. To answer these questions the authors are currently performing a prospective study in patients with postmastectomy TMG flap breast reconstruction.

## Conclusion

The TMG flap presents a suitable option to provide sufficient volume for unilateral and bilateral autologous breast reconstructions and demonstrates low-donor site morbidity, in slim-to-normal weight patients in particular. However, patients should be informed about the likelihood of secondary refinement procedures on the donor site and the possibility of lipofilling to the breast to optimize shape and volume.

## Data Availability

The datasets analyzed during the current study are not publicly available due to patient confidentiality but are available from the corresponding author on reasonable request.
